# One-stage thyroid cartilage laryngotracheal reconstruction for children less than one year old with congenital subglottic stenosis

**DOI:** 10.1017/S0022215124000653

**Published:** 2024-10

**Authors:** Chao Chen, Yi-hua Ni, Le-tian Tan, Yue Huang, Zheng-min Xu

**Affiliations:** Department of Otolaryngology – Head and Neck Surgery, Children's Hospital of Fudan University, 399 Wan Yuan Road, Shanghai, 201102, PR China

**Keywords:** Laryngostenosis, infant, thyroid cartilage

## Abstract

**Objectives:**

To evaluate one-stage thyroid cartilage laryngotracheal reconstruction in children less than one year of age with congenital subglottic stenosis.

**Methods:**

Congenital subglottic stenosis children less than one year old who underwent one-stage thyroid cartilage laryngotracheal reconstruction between 2016 and 2020 in our department were retrospectively reviewed. Their clinical characteristics, treatments and prognoses were assessed.

**Results:**

Eleven congenital subglottic stenosis children (6–11 months) were included: seven with Myer–Cotton grade II, and four with Myer–Cotton grade III. Their tracheal diameters were corrected to normal size using thyroid cartilage, and they were intubated under sedation for two weeks after surgery. Moreover, all of them received anti-infection and anti-reflux therapies during hospitalisation. No breathing difficulty, aspiration, hoarseness or laryngitis was observed during the follow-up period (10–30 months), and their growth and development were age appropriate.

**Conclusion:**

The one-stage thyroid cartilage laryngotracheal reconstruction is a good treatment option for congenital subglottic stenosis children less than one year old with Myer–Cotton grade II–III.

## Introduction

Subglottic stenosis is a narrowing of the airway below the vocal folds and above the trachea. It is a common cause of airway obstruction in children, with a diameter of less than 4.0 mm in newborns and less than 3.0 mm in premature infants.^1^ This disease can be either congenital or acquired. Congenital subglottic stenosis ranks as the third leading cause of airway malformation in children, following laryngomalacia and vocal fold paralysis.^2^ Congenital subglottic stenosis can negatively affect the growth and development of children and, in severe cases, it can lead to life-threatening breathing difficulty and other more pronounced symptoms in infants and young children.^3^

The management of congenital subglottic stenosis is one of the most challenging clinical problems in paediatric otolaryngology. Tracheostomy is an option for relieving acute respiratory distress or securing the airway prior to surgical intervention.^4^ Surgical approaches can be subdivided into endoscopic and open approaches. The endoscopic surgical approaches, applicable to many limited-airway lesions, are less invasive with shorter healing time than the open surgical approaches (e.g. a soft stenosis that may be amenable to an endoscopic procedure). When the stenosis is a firm, matured and cartilaginous framework, we would opt for an open surgical approach.^3^ Open surgical approaches include laryngotracheoplasty, anterior cricoid split, laryngotracheal reconstruction and cricotracheal resection.^5–9^

Congenital subglottic stenosis is caused by failure of laryngeal recanalisation during embryogenesis. It represents more of a hyaline cartilaginous defect than the fibrous scar tissue, which is crucial for determining the surgical repair strategy.^10^ Tracheostomy was the main treatment option for congenital subglottic stenosis until the1970s; however, long-term tracheostomy can cause a series of problems, significantly affecting quality of life and increasing economic burden. Moreover, since the clinical symptoms and related complications in some children cannot be improved with growth, early surgical intervention is recommended. Traditional laryngotracheal reconstruction has thus remained the mainstay treatment strategy for children with severe subglottic stenosis, especially those with serious stenosis scar and cartilage hyperplasia.^1^

In 1974, Fearon and Cotton first reported laryngotracheal reconstruction to treat subglottic stenosis using autologous cartilage grafts in young children.^4^ Autologous materials for enlarging the laryngeal airway commonly include the costal cartilage, auricle cartilage, and nasal septum cartilage, each with its merits and demerits. In 1985, Fry *et al*.^11^ first used thyroid cartilage as an autologous material for laryngotracheal reconstruction. Subsequently, Fraga *et al*.^12^ and Fayoux *et al*.^13^ conducted a series of clinical studies that proved the effectiveness of this practice. In this retrospective study, we report the efficacy and experience of thyroid cartilage application in laryngotracheal reconstruction in eleven children less than one year old with congenital subglottic stenosis.

## Methods

### Subjects and data collection

This study enrolled eleven infants (children 6–11 months old) diagnosed with congenital subglottic stenosis between 2017 and 2020 at the Department of Otolaryngology – Head and Neck Surgery, Children's Hospital of Fudan University. Indications for thyroid cartilage laryngotracheal reconstruction included severe breathing difficulty (Myer–Cotton grade II or III) and feeding difficulty. Children with severe laryngomalacia, vocal fold paralysis, or other laryngeal deformities were excluded. Apart from experiencing severe breathing difficulties, all children faced feeding difficulties and weighed 5.4–8.4 kg, with five of them exhibiting retarded growth (2σ below the mean normal weight). The clinical characteristics of the children are presented in Table 1. The data were derived from medical records, including information on age, gender, clinical symptoms and treatments. The study was approved by the Children's Hospital of Fudan University's institutional review board.

**Table 1.** Clinical characteristics of the congenital subglottic stenosis children (*n* = 11)

	Age (months)	Gender	Weight (Kg)	Myer–Cotton	Surgery history	Clinical symptoms
1	8	Female	6.2	III	None	Breathing difficulty, feeding difficulty, growth retardation
2	7	Male	5.8	III	None	Breathing difficulty, feeding difficulty, growth retardation
3	10	Female	8.1	II	Surgery for congenital heart disease, tracheostomy	Breathing difficulty, feeding difficulty
4	9	Male	5.6	III	None	Breathing difficulty, feeding difficulty, growth retardation
5	11	Male	8.1	II	tracheostomy	Breathing difficulty, feeding difficulty
6	9	Male	8.4	II	None	Breathing difficulty, feeding difficulty
7	10	Male	7.2	II	None	Breathing difficulty, feeding difficulty, growth retardation
8	9	Female	5.4	II	None	Repeated diagnosis of laryngitis, feeding difficulty, growth retardation
9	6	Male	5.5	II	None	Growth retardation, breathing difficulty, feeding difficulty
10	8	Male	6.2	II	None	Breathing difficulty, feeding difficulty
11	9	Female	5.5	III	Tracheostomy	Breathing difficulty, feeding difficulty

### 2.2 Evaluations

The Myer–Cotton grade was used to estimate the severity of stenosis as follows: grade I, ≤50 per cent obstruction; grade II, 51–70 per cent obstruction; grade III, 71–99 per cent obstruction; grade IV, absence of a detectable lumen or complete stenosis. The Myer–Cotton grading was performed using rigid tracheoscopy under general anaesthesia while maintaining spontaneous breathing.

Aspiration was evaluated using a flexible endoscopic evaluation of swallowing. A φ2.9-mm flexible laryngoscope (Olympus Corporation, Tokyo, Japan) was passed through the nasal cavity and into the oropharynx to visualise the laryngopharynx structures. The children were placed in a reclined seating position and were given a methylene blue–water mixture. The following were then observed in the children: the swallowing movement, coverage of the epiglottis after swallowing, whether methylene blue entered the glottis or the subglottis, whether the children coughed during feeding, and whether there was food residue in the larynx and pharynx. The subglottic presence of methylene blue was regarded as aspiration (+), whereas absence of methylene blue under the glottis was regarded as aspiration (−).

The children's prognostic outcomes were evaluated, following the International Paediatric Otolaryngology Group consensus recommendations.^14^ Briefly, after extubation, tracheal tube diameter was evaluated using tracheoscopy and the Myer–Cotton grade system. The thyroid cartilage laryngotracheal reconstruction was considered effective if the child did not have breathing difficulty, even in an active state with a Myer–Cotton grade of less than I.

## 3. Results

### 3.1 Thyroid cartilage laryngotracheal reconstruction

**Figure 1.**
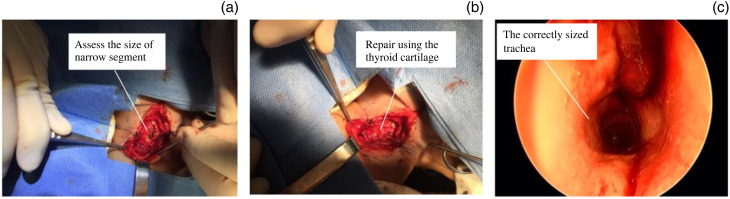
(A) Assessment of length and width of the narrow segment. (B) Stenosis repair using thyroid cartilage. (C) Correctly sized trachea under bronchoscope.

The children were placed in a supine position, and tracheal intubation was performed using a φ2.5-mm tube while spontaneous breathing was maintained. Anaesthesia and ventilation were administered through a temporary tracheostomy. The cricoid cartilage was exposed, the band muscle was separated and the cricoid cartilage was incised. Subsequently, a φ4.0-mm tube was inserted into the trachea through the oropharynx to determine the length and width of the narrowed segment (Figure 1A). This measurement was used to select an appropriately sized fusiform thyroid cartilage. The thyroid cartilage was then implanted into the stenosis using a 5-0 polydioxanone suture (Figure 1B). Finally, the temporary tracheostomy was closed, and the correctly sized trachea was observed using a bronchoscope (Figure 1C). The surgically obtained thyroid cartilage was 9.0 mm long and 5.0 mm wide, which was sufficient to correct the narrowing (average 6.0–8.0 mm long and 3.5–5.0 mm wide) (Table 2).

**Table 2.** Age, weight, Myer–Cotton grade and required size of thyroid cartilage of the congenital subglottic stenosis infants

	1	2	3	4	5	6	7	8	9	10	11
**Age (months)**	8	7	10	9	11	9	10	9	6	8	9
**Weight (kg)**	6.2	5.8	8.1	5.6	8.1	8.4	7.2	5.4	5.5	6.2	5.5
**Myer–Cotton**	III	III	II	III	II	II	II	II	II	II	III
**Width (mm)**	3.5	3.5	4.5	4.0	5.0	4.0	4.5	4.0	3.0	3.0	4.0
**Length (mm)**	6.0	7.0	8.0	7.0	8.0	7.0	8.0	8.0	8.0	8.0	8.0

### 3.2 Post-operative treatment

After surgery, the children remained intubated under sedation for two weeks. They then received intravenous antibiotics for anti-infection therapy and proton pump inhibitors for anti-reflux therapy. Additionally, their necks were immobilised with braces. One week after surgery, the children underwent tracheoscopy and had their tracheal tubes replaced. Tracheal intubation was removed two weeks after surgery, but oral treatment with proton pump inhibitor continued for 2–4 weeks during hospitalisation.

### 3.3 Outcomes and follow up

**Figure 2.**
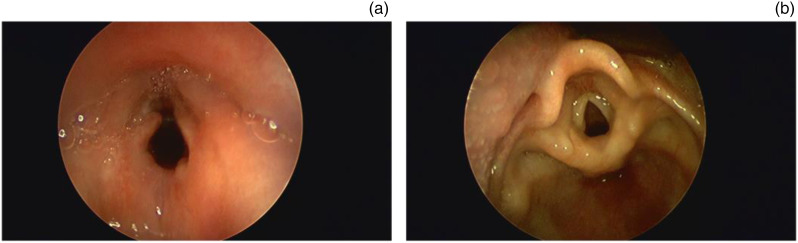
(A) Inflammatory subglottic granulation in glottis two weeks after surgery. (B) Complete remission of inflammatory subglottic granuloma after nebuliser therapy for two weeks.

The tracheal diameters in all the children were corrected to normal size. None of the children had breathing difficulties after surgery, but all had fever one week after surgery. Moreover, all children developed inflammatory subglottic granuloma at two weeks post-surgery, and consequently underwent nebuliser therapy (1 mg budesonide twice daily). Two weeks after the treatment was administered, the granuloma formation was in complete remission (Figure 2).

Eleven children underwent anti-reflux therapy (proton pump inhibitors) for 3–6 months after discharge. The follow-up period was 10–30 months (median: 16 months). No breathing difficulty was observed in any of the children, and their growth and development were age appropriate. Additionally, no child had aspiration, hoarseness, or laryngitis or had to undergo secondary procedures after the reconstruction.

## 4. Discussion

The most common clinical symptoms of congenital subglottic stenosis include breathing difficulty and respiratory infection. However, infants with congenital subglottic stenosis may appear relatively inactive after birth, and significant clinical symptoms would emerge around 6–7 months after birth. In this study, although four children did not show signs of severe breathing difficulty immediately after birth, they had reduced milk intake compared to normal infants, which may have resulted in their eventual significant developmental retardation and lower body weight. These children thus required active intervention to improve quality of life and prevent serious complications.^4^

The primary treatment for subglottic stenosis is surgical intervention, which includes traditional tracheostomy, endoscopic and open surgical approaches.^5–9^ Though tracheostomy is widely recognised as the most effective treatment for congenital subglottic stenosis, long-term use of tracheostomy may affect quality of life of infants and impose a substantial burden on caregivers.^15^ In recent years, endoscopic balloon dilatation has shown a response rate of 70–90 per cent for subglottic stenosis, and a soft stenosis of the airway may be amenable to high-pressure balloon dilatation.^16^ However, this practice does not play a role in the management of congenital subglottic stenosis (except in the extremely rare cases involving very thin web). Dilatation of a congenitally small cricoid cartilage may entail certain risks, for example, rupture.^3^ Laryngeal tracheoplasty using cartilage has thus remained to be an effective treatment for congenital subglottic stenosis.

Cartilage scaffolds can be obtained from various sites, such as the auricular cartilage, hyoid bone, thyroid cartilage and ribs. Among these options, costal cartilage is commonly used because of its easy availability and rigid structural support. For infants with congenital subglottic stenosis, the required scaffold typically measures approximately 3.5 to 5.0 mm in width and 6.0 to 8.0 mm in length. Thyroid cartilage offers a broader scaffold range, measuring up to 5.0 mm in width and 9.0 mm in length, making it a suitable material.^17^

Nguyen *et al*.^18^ conducted a study comparing the use of costal and thyroid cartilages in laryngotracheal reconstruction in children. It was determined that although there was no significant difference in extubation rates between the costal and thyroid cartilage group, the costal cartilage group experienced prolonged surgical procedures and occasional complications in the second surgical field. Additionally, post-operative pain at the donor site (costal cartilage) significantly increased the peri-operative nursing burden and respiratory complications.

One drawback of using thyroid cartilage compared to costal cartilage is its limited length for widening the trachea. Therefore, if a child requires a larger cartilage scaffold, costal cartilage should be chosen. Although there are limited reports on young infants, it is hypothesised that the optimal indications for thyroid cartilage laryngotracheal reconstruction include Myer–Cotton grades II and III, with length not exceeding the second tracheal cartilage ring, and rigid cartilage support on the lateral wall of the narrowed segment.

Laryngotracheal reconstruction is typically performed in one or two stages. The two-stage procedure involves selection of a laryngeal model or implantation of a T-tube for airway support, which requires secondary extubation after 3–6 months or longer with the tube. In contrast, the one-stage approach uses tracheal intubation for only approximately two weeks after the operation, followed by extubation.^19^ In our study, all children underwent one-stage surgery, and were extubated two weeks after tracheal intubation. This approach can prevent feeding and nursing problems associated with prolonged tracheal T-tube implantation, thereby reducing the risk of respiratory tract infections. However, since one-stage surgery may increase the post-operative monitoring and nursing burden, tracheal intubation must be replaced one week after surgery to avoid intubation-induced infection. Furthermore, sedation and analgesia two weeks after surgery can be administered to minimise the possibility of displacement and recurrence of secondary wound stenosis.^20^

- Traditional laryngotracheal reconstruction remains the main treatment strategy for infants with severe subglottic stenosis
- Cartilage scaffolds can be obtained from a variety of sources, including auricular cartilage, hyoid bone, thyroid alar cartilage, and ribs
- Thyroid alar cartilage is simpler and more convenient than other cartilage materials
- Indications of one-stage thyroid alar cartilage laryngotracheal reconstruction are Cotton-Myer II, stenosis III, the length not exceeding the 2nd tracheal cartilage ring, and the rigid cartilage support on the lateral wall of the narrow segment
- One-stage thyroid alar cartilage laryngotracheal reconstruction requires high demands on post-operative monitoring, nursing, and replacing the tracheal intubation one week after surgery to avoid intubation-related infection
- Sedation and analgesia two weeks after surgery may avoid displacement and recurrence of secondary stenosis of the wound

In the present study, inflammatory subglottic granulation was observed in all children due to intubation. Use of steroid was avoided to prevent potential impact on wound healing two weeks after surgery. To ameliorate the granulation tissue, 1 mg of budesonide was administered orally via PARI nebuliser (PARI Business Group, Starnberg, Moosstraße 3, Germany) two weeks after surgery. The follow up performed with laryngoscopy one month post-surgery revealed complete remission of the granuloma formation. Moreover, all children in this study experienced fever for 3–7 days after surgery, with peak temperatures of 37.8–39.0°C. In three cases, increased infection indicators were observed (increased white blood cells and procalcitonin), but all of them improved with cefoperazone/sulbactam anti-infective treatment. In other cases, onset of fever occurred 1–3 days post-surgery, and it then gradually subsided to normal body temperature within 5–7 days post-operation without any abnormalities detected in subsequent tests. These observed symptoms are interpreted as stress-induced post-operative fever reactions.

In conclusion, one-stage laryngotracheal reconstruction using thyroid cartilage appears to be a good treatment option for eligible children with congenital subglottic stenosis. Thyroid cartilage is readily accessible for trachea dilatation and offers a simpler and more convenient alternative to other cartilage materials. Given that this procedure is still in its nascent stage among children in China, its safety and feasibility need to be studied further.
